# Mind Altered, or calmed down: the enigma of substance use and psychosis

**DOI:** 10.1192/j.eurpsy.2025.145

**Published:** 2025-08-26

**Authors:** A. Batalla

**Affiliations:** UMC Utrecht, Utrecht, Netherlands

## Abstract

**Abstract:**

Cannabis use is highly prevalent among individuals at risk for psychosis, yet its role remains paradoxical—offering both temporary relief and potential harm. In this session, we present preliminary results from a study examining cannabis perception, use patterns, and motivations in cannabis users with and without psychosis. Findings reveal that cannabis users with psychosis consume over three times more THC but do not perceive significantly greater risks. They also exhibit a higher risk of cannabis addiction, which may influence their risk assessment and experience of cannabis effects. Both groups reported similar reasons for use and quitting, though individuals with psychosis were more likely to cite cost as a reason for quitting. These insights suggest that financial concerns could be a target for intervention. Understanding why individuals vulnerable to psychosis turn to substances is crucial for refining treatment strategies, as addressing these underlying motivations may enhance therapeutic outcomes.

**Disclosure of Interest:**

None Declared

